# Supplementary material for: Chromothripsis during telomere crisis is independent of NHEJ, and consistent with a replicative origin
Source: Genome Res. 2019 May;29(5):737–49. doi: 10.1101/gr.240705.118 (PMC6499312; doi:10.1101/gr.240705.118)
Supplement: Supplemental Material [file supp_gr.240705.118_Supplemental_file_1.zip › contigs/annotated_contigs/DB55/contig.2.DB55_length_711_mean_cov_5.9746835443.docx]

**DB55_length_711_mean_cov_5.9746835443**

```
ATCCTACTGAAACTGTTCCAAAATATTGAAGAGGAGGAACTCTTTGCTAACTCATTATATGAGGCCAGCATTATTCTGATACCAAAACT
 >chr9:8151268-8151644 + E=2e-214 p=3e-03
TGGCAGAGACACACAAAAAAAGAAAACTACAGGCCAATATCCCTTATGAACATAGAAATAAAAATCCTCAACAAAATAGTAGCAAATCA

AATCCAGCAGCACATCAAAAAACTAATCAAACATGGTCAAGTAGGCTTTTTTTCCAGGAATCCAAGGTTGGTTGAACATATGCAAATCA

ATAAATGTGATTGATCACATAAACCGAACTAAAAACAAAAGCCATATGATTATCTCAATAAATGCAGAAAAGGCTTTCAACAAAATTCA

ATATCCCTTCATGTTAAAAA|TATAGTAAAATATAGTA|AAATATAGTAAAAAGGCTGCAAAAGATGTGTCCAGTCTTATCCTCCACCC
                                      >chr9:8205324-8205613 + E=5e-147
AAGTCTGCCCCACCTTTACTCCATTGACTGTTTCAGGTATGACATGAAATTATGTCTCAAACTGCATTAGAATGGACTGTGTAATGCAT

AGATATTAATATTTTGGTCTTGGATTTTCAGTACAAGCCTCTCTACCTCCCATAAGCCTCACAGGCAACGGCAGGTTGCATCCTTAAGG

ACGTGCATTAGATGACCATGTCTTTGTTTCTCTTATTCTCTTGTTTCATTTGTTTCCTTTT|TATTTTTGTAGTCAATATGATTTTTAA

AT
```
